# From feedback to action: a process evaluation of implementation strategies for sepsis bundles in emergency departments

**DOI:** 10.3389/fmed.2026.1748494

**Published:** 2026-02-05

**Authors:** Jacqueline F. Hayes, Hannah E. Frank, Aden Littlewood, Linda E. Guzman, Kathleen M. Terry, Christa Schorr, David Portelli, Gary Phillips, Lori Harmon, Jessyca Goldstein, Laura Evans, R. Phillip Dellinger, Mitchell M. Levy

**Affiliations:** 1 Department of Psychiatry and Human Behavior, The Warren Alpert Medical School of Brown University, Providence, RI, United States; 2 Weight Control and Diabetes Research Center, The Miriam Hospital, Providence, RI, United States; 3 Lightning Strategies LLC, Northport, NY, United States; 4 Cooper Research Institute – Critical Care, Cooper University Healthcare, Camden, NJ, United States; 5 Department of Emergency Medicine, The Warren Alpert Medical School of Brown University, Providence, RI, United States; 6 Center for Biostatistics, The Ohio State University, Marion, OH, United States; 7 Society of Critical Care Medicine, Mount Prospect, IL, United States; 8 Department of Pulmonary, Critical Care and Sleep Medicine, Warren Alpert Medical School at Brown University, Providence, RI, United States; 9 Division of Pulmonary, Critical Care and Sleep Medicine, University of Washington, Seattle, WA, United States; 10 Critical Care Division, Cooper University Health Care, Cooper Medical School of Rowan University, Camden, NJ, United States

**Keywords:** emergency medicine, process evaluation, sepsis, sepsis bundles, septic shock

## Abstract

**Background:**

Sepsis is a burdensome and costly condition and a leading cause of death in acute care centers. Guided by the Exploration, Preparation, Implementation, Sustainment (EPIS) Framework, the Assessment of Implementation Methods in Sepsis (AIMS) study is an ongoing hybrid type 2 effectiveness-implementation study. One co-primary aim is to compare two multi-component sepsis “bundles”—one accomplished within 3-h and one within 1-h—and their effects on mortality and related health outcomes. The other co-primary aim is to assess implementation strategies that support bundle implementation within emergency departments. Implementation strategies include learning collaboratives, provision of educational materials, audit and feedback reports, capturing and sharing local knowledge, and technical assistance. The goal of this implementation-focused process evaluation was to identify barriers and facilitators to the implementation process and to develop subsequent adaptations to enhance implementation.

**Methods:**

A multi-method data collection and analysis was undertaken in the Implementation stage. The two champions (one nurse and one physician) from each of the 18 AIMS study sites were invited to participate in semi-structured interviews. Learning collaborative attendees completed quantitative satisfaction surveys. After analysis, potentially impactful and feasible modifications to the implementation process were identified, documented using the FRAME-IS, and enacted.

**Results:**

Synthesis of 24 interviews and 19 surveys indicated that the implementation strategies were generally executed as planned and respondents were satisfied with the implementation process. Monthly learning collaboratives included helpful topics and facilitated inter-site networking and learning. Educational materials were valuable resources for onboarding and ongoing reference, and monthly audit and feedback reports helped to quantify progress and benchmark with other AIMS sites. Barriers and related adaptations were focused on simplifying and streamlining touchpoints and materials, further supporting inter-site networking and learning, and increasing knowledge of and access to resources. Fifteen adaptations (content = 7; context = 4; evaluation = 2; training = 1; and multi-purpose = 1) were made to increase the acceptability, appropriateness, or feasibility of the implementation effort (*n* = 12), improve fidelity to bundles (*n* = 2), and to increase adoption of bundles (*n* = 1).

**Conclusion:**

The implementation strategies were well-received and site-specific feedback led to modifications. The summative evaluation will provide insight into if and how modifications enhanced implementation efforts.

**Trial registration:**

ClinicalTrails.gov, Identifier NCT05491941.

## Introduction

Sepsis is a burdensome and costly condition that affects 1.7 million individuals in the US each year and results in 350,000 deaths (1). Within intensive care units, sepsis is the leading cause of admission and a leading cause of death (2). Sepsis bundles are a discrete set of intervention components to guide sepsis clinical care, including measuring lactate, obtaining blood cultures, administering antibiotics, and beginning fluid resuscitation if needed. A large body of literature demonstrates a link between sepsis bundle utilization and improved patient outcomes, with higher compliance associated with lower mortality (3–5). The 3-h bundle specifies that all bundle elements should be initiated within 3 h. Recent evidence suggests that initiation of all bundle elements within a 1-h timeline can improve patient outcomes and reduce mortality (6, 7); however, the hour-1 bundle (8) has not been widely adopted within emergency departments. Ensuring widespread early adoption of sepsis bundles in emergency departments is critical to improve survival (9–11).

The Assessment of Implementation Methods in Sepsis (AIMS) study is a Hybrid 2 effectiveness-implementation trial designed to test the effectiveness of the hour-1 bundle against the standard 3-h sepsis bundle on mortality and other secondary outcomes at 18 emergency department sites in the United States (12). The co-primary implementation aim is to examine the use of a set of 10 tailored implementation strategies on fidelity to both bundles within the specified time frames. The study follows the Exploration, Preparation, Implementation, Sustainment (EPIS) Framework, a process implementation framework that delineates stages of implementation and contextualizes the outer context, inner context, bridging factors, and the evidence-based innovation (13). Per the protocol (12), a process evaluation was planned to take place at the mid-point, or 15 months into, the Implementation phase of the study. The results of the process evaluation are featured in this report.

An implementation-focused process evaluation is designed to identify discrepancies between the implementation plan and enactment of the plan, including identification of ongoing or unanticipated barriers (14, 15). This provides an opportunity to tailor planned implementation strategies to support the implementation effort. Process evaluations in acute hospital settings are relatively rare (16) and often occur at the conclusion of an implementation effort (17), making it impossible to respond in real time and modify the implementation approach as needed. The current paper addresses both of these gaps in the literature.

The aims of this implementation-focused process evaluation were to: (1) identify barriers and facilitators to the execution of the implementation process; and (2) to adapt or modify implementation strategies to improve implementation of the assigned sepsis bundle arm (hour-1 vs. 3-h). Identification of barriers and subsequent modifications to the implementation strategies within this Hybrid 2 effectiveness-implementation trial were deemed necessary to ensure the ability to draw firm conclusions regarding the effectiveness outcomes. Adaptations of implementation strategies were tracked with the Framework for Reporting Adaptations and Modifications to Evidence-based Implementation Strategies (FRAME-IS), which offers a standardized method to catalog changes made to implementation strategies and promotes the ability to understand how implementation and effectiveness outcomes may change as a result of enacted modifications (18).

## Methods

### AIMS study implementation strategy overview

To achieve the goals of the AIMS study, two site champions—one physician and one nurse—were identified at each study site. The site champions function as the primary site contacts for the study and are responsible for overseeing the implementation of the assigned bundle at their site. In addition to *identifying champions,* we *assessed for readiness with a formative evaluation* (19), and planned to *re-examine implementation* with: (a) this process and (b) a future summative evaluation. Beyond these four strategies, six other Expert Recommendations for Implementing Change (ERIC) implementation strategies were selected to target fidelity to the assigned sepsis bundles in the emergency departments of 18 hospital sites (20). First and second, we established ongoing *learning collaboratives* through which we provide *ongoing training*. These are monthly virtual meetings offered to all providers and staff within sites randomized to the same bundle arm. Each learning collaborative meeting was focused on a key topic, informed by the developmental formative evaluation (19), and typically included didactic and/or interactive components. In-person meetings with site champions and the Steering Committee took place as an extension of the learning collaboratives and happened at the beginning and middle of the study. Access to a message board hosted on the Society of Critical Care Medicine (SCCM) Connect website was also considered part of the learning collaboratives, as participants could review learning collaborative materials (recordings, slides) and ask or respond to questions via this platform. Third, we *developed and distributed educational materials* focused on the sepsis bundle components. These materials were designed to address frequent issues that arose with implementation and were provided at the beginning and throughout the study. Fourth, sites received monthly *audit and feedback* of their adherence to bundles via reports sent by email to the site champions and data abstractors. Meetings with the implementation science team were available to sites upon request to aid in site-specific tailoring on bundle implementation based on audit and feedback reports. Fifth, we developed and implemented *tools for quality monitoring* through ongoing training of data abstractors at each site. Finally, we supported *changes to record systems* (i.e., Epic, the electronic health record system) through coaching to sites about ways to more easily abstract data from Epic for the study and to modify Epic order sets and alerts.

### Process evaluation overview

**Figure 1:**
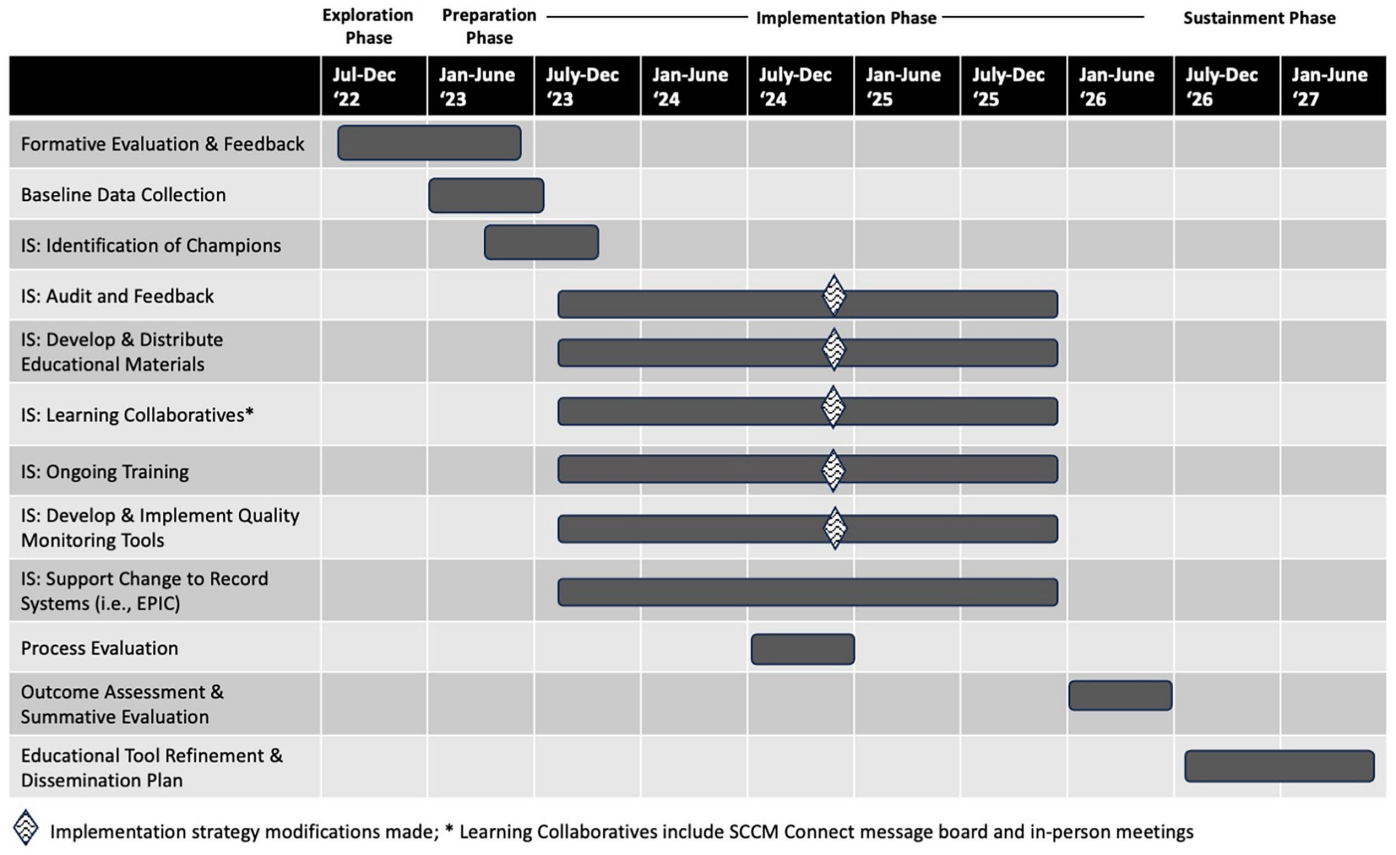
AIMS study timeline of implementation strategies and constructs.

The process evaluation occurred at the mid-point of the 30-month implementation effort (in July–August 2024; see Figure 1) and a multi-method data collection process was used. Semi-structured qualitative interviews assessed how implementation efforts have supported bundle implementation, ways to improve the implementation process, and barriers not being addressed. Two sources of quantitative data were collected: (1) learning collaborative satisfaction and (2) usefulness of activities offered at the mid-study in-person meeting. Following analysis of qualitative interviews and learning collaborative surveys, the implementation team and AIMS Study Steering Committee identified and enacted next steps to modify implementation strategies and address ongoing barriers. The overall goal was to understand and address barriers and facilitators of fidelity to the assigned bundle, the primary implementation outcome. Of note, the original plan for the process evaluation was to review recorded learning collaboratives for themes (12); however, given that learning collaboratives often included significant didactic components and were narrow in their scope (i.e., would primarily provide information on the learning collaborative experience), the alternative process specified above was agreed upon by the implementation team as a way to obtain broader feedback on implementation challenges and successes to date. Effectiveness outcomes were not included in this implementation-focused process evaluation as interim analyses could compromise study integrity (21). For similar reasons, fidelity was not aggregated and reviewed; however, monthly creation and review of audit and feedback reports indicated room for improvement in bundle adherence across sites. A manuscript presenting the primary outcomes will be published at the conclusion of the trial.

### Data collection and analysis

A semi-structured guide was created by the implementation team for the interviews. The guide asked about: (1) perceptions of the learning collaboratives; (2) ways in which the study had supported bundle implementation; (3) ways in which support could be improved; (4) ongoing barriers not currently being addressed; and (5) desired outcomes from the mid-study in-person meeting. The nurse and physician champions from each site were invited to participate in a virtual interview held on a video conference platform. A total of 24 qualitative interviews were conducted with nurse and physician champions from 17 out of the 18 sites (one site did not respond to our requests to schedule an interview). Although saturation, or the lack of novel data being collected, was observed after 12 interviews, we continued to conduct interviews until we had at least one representative from the 17 sites who responded to our outreach. Interviews lasted approximately 20 min. Rapid qualitative analysis procedures (22, 23) were used given the tight turnaround to inform the in-person meeting agenda and changes to implementation strategies. Coders (HEF, AL, LEG, JFH) met and developed a structured summary template that was used to code individual interviews. Two interviews were coded by the analysis team together to ensure coding consistency and to revise the summary template as needed. The remainder of the interviews were split up and summarized by individual coders. All coding questions were reviewed in weekly coder team meetings. Once complete, the interview summaries were input into a matrix with interview questions as columns and respondents as rows. All coders collectively and collaboratively reviewed the matrix and identified themes by column. In addition to the interview guide questions that asked about learning collaboratives and the upcoming mid-point in-person meeting, two other implementation strategies were repeatedly mentioned in the interviews in response to probes (i.e., audit and feedback and educational materials). Thus, themes were re-organized by implementation strategy after initial analysis of responses in the matrix.

Quantitative surveys regarding perceptions of the learning collaboratives were collected at the end of the August 2024 learning collaborative from attendees (*N* = 19 respondents). Surveys included 6-items participants rated on a 7-point Likert scale from 1 (strongly disagree) to 7 (strongly agree) regarding satisfaction and appropriateness of the learning collaboratives. Surveys regarding usefulness of the activities offered at the in-person meeting held in December 2024 were collected at the meeting conclusion (*N* = 25 respondents). Surveys asked about how well each in-person meeting activity (e.g., storyboard rounding) supported (1) networking with and (2) learning from other sites. Responses were provided on a 10-point Likert scale with 10 representing the highest perceived usefulness of each activity. Means of each item were averaged to understand how each activity supported networking with and learning from other AIMS sites.

### Action plan creation and implementation

Following review of the results of the quantitative and qualitative data, the implementation team reviewed barriers and identified areas for improvement. One of the coders (AL) created an exhaustive list of all recommendations that arose from qualitative interviews that might be used to address common barriers. Each of these recommendations was reviewed and rated for both feasibility and impact by the implementation team. Recommendations that were considered highly feasible and highly impactful were brought for consideration by the Steering Committee. The Steering Committee approved the recommended changes and worked collectively to operationalize and enact these changes. Changes made to the implementation strategies were tracked with the FRAME-IS by the implementation team (18). The FRAME-IS catalogs modifications made to implementation strategies by: (1) what is modified; (2) the nature of the modification; (3) the primary goal and rationale for the modification; (4) the timing of the modification; (5) participants in the modification decision-making process; and (6) the scope of the modification.

## Results

### Process evaluation themes by implementation strategy

**Audit and Feedback.** Champions indicated that audit and feedback reports were helpful to identify performance trends, optimize practices, and assess the progress of their site in comparison to other hospital sites. Some respondents also reported successfully using these reports to engage hospital leadership, presenting data as evidence to support requests for resources or process changes (e.g., to hire a sepsis coordinator or make changes in Epic). A common concern noted with audit and feedback reports were that monthly reports were not frequent enough to be actionable, particularly if they had a more comprehensive internal process for audit and feedback. Others stated that reports were too complex and often led to confusion, and that uploading data for the reports was slow, burdensome, and time-consuming, reducing staff motivation to participate. Champions suggested focusing reports only on key metrics, such as compliance and mortality, clarifying what patients are included in data, and providing more tailored feedback at the site and/or individual staff level. Respondents were generally unaware that meetings with the implementation team were available to review audit and feedback reports, and no respondent indicated utilizing this offering.

**Develop and Distribute Educational Materials.** Champions reported that the visual educational materials, such as a diagram of the sepsis pathway, were the most utilized resources as they helped organize thinking and could be easily referenced. There were no barriers to use indicated, but participants suggested providing more concise educational content, such as short videos on sepsis concepts.

**Learning Collaboratives**. Participants appreciated opportunities in the learning collaboratives to engage with and learn from other sites, particularly through interactive activities. They also described enjoying the diversity of topics and included multiple provider groups (e.g., physicians and nurses). The learning collaboratives were viewed to be most helpful when focused on practical solutions and overcoming barriers; topics related to Epic sepsis workflows, causal pathway diagrams, and fluid requirements were particularly favored. For barriers, time constraints limited participants’ abilities to engage with learning collaboratives. While the meetings were recorded and available for viewing, recordings were considered to be less engaging than attending live. Champions also indicated that some learning collaborative topics (e.g., need for fluid resuscitation) were viewed as too basic or redundant, limiting their appeal to more seasoned clinicians. There was also mixed feedback on some components, with some respondents indicating that learning collaboratives focused on presentations from participating sites, staff turnover, and specific case examples were helpful and relevant, while others found them to be irrelevant to their position or context. For example, hearing about experiences of a highly resourced site (e.g., with multiple funded roles to support sepsis bundle implementation) was often perceived as unhelpful for less-resourced sites. There was also some disagreement around frequency of meetings, with some appreciating the consistent monthly offering and others indicating that learning collaboratives took up too much time. Suggested modifications from respondents were to provide discussion topics and the opportunity to submit questions ahead of the learning collaborative. They also identified a desire for practical and actionable takeaways from each meeting, as well as resources and materials for sites to use locally. Quantitative data from the learning collaborative survey (*n* = 19) indicated high satisfaction with the learning collaboratives overall, with means of at least 6 out of a possible 7 on all queries (Table 1).

**Table 1:** Results of the learning collaborative satisfaction survey (*n* = 19).

Item	M +/− SD
I am satisfied with the format of the AIMS Learning Collaborative.	6.4 ± 0.8
I find the AIMS Learning Collaborative to be applicable to my role.	6.5 ± 0.6
I am satisfied with the trainers who have led the AIMS Learning Collaboratives.	6.6 ± 0.6
I am satisfied with my overall experience with the AIMS Learning Collaborative.	6.4 ± 0.7
The balance between presentations, discussion, and activities fits my style of learning.	6.4 ± 0.7
I would recommend the AIMS Learning collaborative to others.	6.4 ± 0.7

Responses range from 1 (strongly disagree) to 7 (strongly agree).

***SCCM Connect*.** Respondents noted that the SCCM Connect library was initially helpful for answering questions and accessing resources related to the learning collaboratives. While many noted that their SCCM connect usage decreased over time as familiarity with the study increased, it remained a valuable reference for some. Technical challenges, including logging in, slow or limited responses from peers, difficulties using the platform on a mobile device, and time constraints were noted as barriers to using the site. Participants requested user-friendly technological improvements, a resource that provided an overview of SCCM functions, and structured discussions on the message board.

***In-person Meeting***. Champions reported that they appreciated the chance to connect, collaborate, and network with other sites at the initial in-person meeting and found interactive and small group activities helped to create a sense of community. However, some respondents noted that they initially had felt concerns that they would not be performing on par with other AIMS sites. The respondents had several suggestions for the upcoming mid-study in-person meeting, including hearing about and sharing successes among sites without promoting competitiveness and expanding discussions and interactive activities where sites can share and learn from other sites’ experiences. Requests were also made for a debrief of the meeting to be shared for individuals who would be unable to attend.

**Cross-Cutting Barriers to Sepsis Bundle Compliance.** A primary barrier to bundle adherence noted by respondents was that identifying sepsis in complex patients or patients who present with atypical symptoms leads to difficulties in diagnosis and slows the implementation of bundles. Additionally, participants described heavy workloads and competing priorities that interfered with full engagement with sepsis protocols and educational and training initiatives. Epic (EHR) issues were also noted as ongoing barriers, including data extraction from outdated data systems, alert fatigue and desensitization to sepsis-related alerts, and software updates that negatively impact upgraded Epic sepsis processes. There were also several technological barriers related to engaging with some of the implementation strategies, including firewalls and software restrictions that made it difficult to access online resources such as SCCM Connect. Leadership and bureaucratic challenges were reported, with some sites reporting difficulty gaining leadership buy-in, which inhibited interest in and approval of sepsis care improvements to support bundle implementation. This seemed to be particularly challenging within larger hospital networks in which decisions proceed through multiple levels of administration. Finally, participants indicated that high turnover rates made it challenging and burdensome to provide sufficient sepsis bundle training and led to inconsistent application of sepsis protocols.

**Cross-Cutting Facilitators to Sepsis Bundle Compliance.** While some sites noted difficulties with leadership, most sites reported increased leadership buy-in largely due to AIMS study participation. With study participation, champions indicated the ability to increase sepsis awareness among leadership and to successfully seek and receive additional resources for bundle implementation, such as a dedicated sepsis coordinator. Overall, they noted a more supportive environment for adopting new sepsis protocols. Participants also reported improvement in inter-organizational environment and networks. They cited valuable learning in their interactions with other sites and a sense of community and collaboration that was a product of participation in AIMS study implementation activities.

### Adaptations to implementation approach

Implementation team synthesis of the qualitative and quantitative data led to the identification of several areas for improvement. As described above, modifications that were rated by the implementation team as highly feasible and likely to have high impact were brought forth to the Steering Committee. At this stage, suggested adaptations to implementation strategies were operationalized and plans for their execution commenced. Table 2 summarizes the final outcomes of this process, which is also described briefly.

**Table 2:** The identified areas for improvement and recommended modifications following the process evaluation and the operationalization of the modifications.

Implementation strategy	Area for improvement*	Recommended modification	Operationalization of modification
Audit & feedback	Clarify limitations of reports to help identify discrepancies between hospital and AIMS study reports and improve their ability to be digested quickly	Add clarification notes to audit and feedback reports (e.g., that it includes patients who are not transfers)	Add language on bottom of audit & feedback reports that clarifies the source and classification of data being provided On report, refer to how the measures apply to AIMS versus CMS and specify that data only includes ED patients Review the reasons for potential discrepancies at in person meeting
	Challenges generating data for reports and resultant delays in receiving feedback	*No solution identified*	N/A
	Increase awareness that meeting with implementation scientist to review reports is an option	Publicize (during learning collaboratives and at in-person meeting) that all sites can schedule one-on-one meetings with the study implementation scientist to go over reports	Announce / remind about availability of meetings with implementation science team
Develop and distribute educational materials	Provide concise educational content	Upload short videos on sepsis concepts that can be shared with others at each site	Educational subcommittee recorded a set of brief (3-min. or less) videos on topics important to users (e.g., flow charts, definitions of sepsis) for dissemination during and after the study and posted on SCCM Connect. Engagement with videos will be tracked for summative assessment
Learning collaboratives: monthly meetings	Increase engagement and relevance of topics	Provide discussion topics for learning collaboratives ahead of time so people can prepare and submit questions More structured meetings with clear agendas, main points at the beginning, and time for questions	A templated agenda was developed for the collaboratives that is the same every time to participants know what to expect (meeting focus; action steps; key takeaways) Topics for next month’s learning collaborative are announced at the end of each meeting and on SCCM connect Key manuscripts/resources from learning collaboratives are being distributed, as applicable
	Provide resources that are relevant for those who cannot attend live and for sites to use locally	Provide a summary that covers what happened in meeting, including key points and relevant articles, to help sites apply findings locally	Video conferencing AI software used to provide meeting summary for distribution following learning collaboratives A key takeaway slide is posted on SCCM Connect
Learning collaboratives: SCCM connect	Improve access to and engagement with SCCM Connect	Add a reference guide (and review in learning collaborative meeting) that shows how to use SCCM connect, including how to reset passwords and who to contact for help	Create a video reference guide to distribute to sites to illustrate how to access materials more easily Post more frequently to encourage engagement
	Provide alternatives to SCCM connect	Create smaller working groups / subgroups centered around specific electronic health record challenges	Generate ideas for future LC topics / small workgroups at in person meeting Use LC meetings to create breakout rooms and generate discussion among smaller working groups
Learning collaboratives: in-person meeting	Address issues of compliance in comparison to other sites, but avoid promoting competitiveness	Distribute audit and feedback reports and review how to interpret them Break out huddles to discuss available ED resources	Review audit and feedback report templates; Introduce Situation, Background, Assessment, and Recommendation (SBAR) tool
	Learn from other sites about methods to overcome continued barriers to improving compliance	Storyboard rounds focused on what has been successful and what is still a challenge	Lead storyboard rounds with emphasis on identifying each site’s strengths
	Create a sense of community by provide opportunities to connect, collaborate, and network with other sites	Create contact list of study PIs and their areas of expertise to improve communication and collaboration Networking breaks throughout the day Small group team building activity	Facilitate networking by creating lists of all attendees, offering unstructured time at lunch for networking, and begin with a small group team building activity
	Debrief on meeting afterward for those who cannot attend	Provide a summary of the meeting with key takeaways for those unable to attend	Send out 1-page summary of in person meeting highlights to all site providers and staff

LC, Learning collaboratives; ED, Emergency department; SCCM, Society of Critical Care Medicine; *For the in-person meetings, this column represents requests made by interviewees for in-person meeting goals and/or activities.

For learning collaboratives, in order to increase engagement with and relevance of topics and to provide resources for those unable to attend live, the discussion topics were publicized 3 months in advance to allow for preparation and question submission. In addition, a template for the structure of the learning collaboratives was developed to guide presenters in their planning and presentation, and summaries including key take away of the meetings began to be distributed. Meetings with the implementation team were advertised at the conclusion of learning collaboratives and at the in-person meeting to increase awareness and promote uptake.

To improve access to and engagement with SCCM Connect, a video-based user guide was created and distributed. Opportunities to connect with other sites about shared topics of interest were also developed by creating breakout rooms with different topics at one of the learning collaborative meetings. Most of the suggestions for the in-person meeting were rated to be feasible and impactful and were ultimately implemented, including increased opportunities for structured and unstructured networking and teambuilding, small group activities that highlighted strengths of each site to reduce competitiveness, and a summary of activities and takeaways for those unable to attend. Attendees at the meeting who completed the post-meeting survey (n = 24) rated the opportunity for unstructured discussion (i.e., breaks and lunch) most supportive of networking and rated storyboard rounding most supportive of learning from other sites, though generally all activities were considered to be supportive of networking and learning (Table 3).

**Table 3:** Results from the in-person meeting post-meeting survey.

Item	M ± SD
Please rate how well each activity supported networking with other sites:	
Distribution and review of audit & feedback reports	8.4 ± 2.0
Breakout groups to discuss available emergency department resources	8.6 ± 1.9
Team-building activity	8.6 ± 2.3
Storyboard rounding	8.5 ± 1.9
Breaks and lunch	9.2 ± 1.4
Creating a contact list of site personnel and areas of expertise	7.2 ± 3.5
Please rate how well each activity supported your learning from other sites:	
Distribution and review of audit & feedback reports	7.8 ± 2.2
Breakout groups to discuss available emergency department resources	8.8 ± 1.7
Team-building activity	7.3 ± 3.3
Storyboard rounding	9.0 ± 1.4
Breaks and lunch	8.4 ± 2.2
Creating a contact list of site personnel and areas of expertise	7.1 ± 3.5

Responses range from 1 (strongly disagree) to 10 (strongly agree).

To supplement existing educational materials and ensure access to concise educational materials, short videos on key sepsis concepts and problem areas were *developed* and *distributed* among sites. Thus, we expanded upon the plan to distribute educational materials and developed additional materials in response to feedback. Additional detail was added to audit and feedback reports to clarify limitations of data and discrepancies between in-house site reports and AIMS study reports. However, no feasible solutions were identified to address site challenges in generating data and resultant delays in receiving feedback. Notably, these activities were difficult for sites but related more to study procedures than to bundle implementation.

Final modifications were cataloged using the FRAME-IS (Table 4). A total of 15 adaptations were documented, with 7 content modifications, 4 context modifications, 2 evaluation modifications, 1 training modification, and 1 modification that was categorized as both a content and a context modification. The primary goals of the modifications were to improve fidelity to bundle implementation (*n* = 2), to increase adoption of bundle implementation (*n* = 1), and to increase the acceptability, appropriateness, or feasibility of the implementation effort (*n* = 12). All modifications occurred in the implementation phase and were planned and enacted by the research team.

**Table 4:** Modifications made to the implementation process during the AIMS process evaluation categorized by the FRAME-IS.

FRAME-IS Question	Modification frequency	
	*n*	%
Module 1: Brief description of EBP		
The implementation strategy being modified is:		
Audit and provide feedback	6	40%
Develop and implement tools for quality monitoring	2	13%
Purposely reexamine the implementation	1	7%
Creating a learning collaborative	5	33%
Develop educational materials	1	7%
Module 2: WHAT is modified?		
What is modified?*		
Content	8	53%
Evaluation	2	13%
Training	1	7%
Context	5	33%
Context modification?*		
Format	3	20%
Population	1	7%
Other: Increased promotion	1	7%
n/a	10	67%
Module 3: What is the NATURE of the content, evaluation, or training modification?*		
Tailoring/tweaking/refining	6	40%
Changes in packaging or materials	1	7%
Removing/skipping elements	2	13%
Substituting	1	7%
Lengthening/ extending (pacing/timing)	1	7%
Repeating elements or modules of the implementation strategy	1	7%
Adding elements	4	27%
Module 4: What is the RATIONALE for the modification?		
What is the goal?		
Improve fidelity to the EBP	2	13%
Increase adoption of the EBP	1	7%
Increase the acceptability, appropriateness, or feasibility of the implementation effort	12	80%
What is the level of the modification?		
Implementer level	14	93%
Organizational level	1	7%
Module 5: WHEN is the modification initiated, and is it PLANNED?		
When is the modification initiated?		
Implementation phase	15	100%
During the implementation phase process evaluation	8	53%
Is modification planned?		
Planned/Proactive	4	27%
Planned/Reactive	11	73%
Module 6: WHO participates in the decision to modify?*		
Researcher	15	100%
Module 7: How WIDESPREAD is the modification?*		
Individual practitioner charged with implementing the EBP	6	40%
Implementation/facilitation team	9	60%
Specific implementer/facilitator	1	7%
Organization	1	7%
Clinic/unit	1	7%

*Categories are not mutually exclusive and modifications may not sum to 100%.

## Discussion

Process evaluations in the midst of an implementation effort allow for identification of differences between the planned and actual implementation, including any ongoing or unexpected obstacles, and provides opportunity to address them. Broadly, we found the implementation strategies, particularly learning collaboratives, to be functioning as planned with respondents reporting high utilization of and satisfaction with them. Nevertheless, several barriers to the implementation process were reported and modifications to the content, context, and evaluation of implementation strategies were made.

Sites seemed to find the learning collaboratives, including the in-person meeting, to be particularly beneficial in bundle implementation. Suggested improvements to the learning collaboratives were primarily around tailoring the topic foci, increasing the structure of the meetings, and providing summaries for individuals unable to attend. Learning collaboratives have been an effective way to promote implementation in healthcare systems (24) and within the AIMS study, they have been carried out primarily in line with best practices (e.g., supporting peer-to-peer learning), likely increasing their satisfaction (25). Modifications made, such as communicating expectations and agendas in advance, served to increase their adherence to best practice recommendations (25). Educational materials were also reported to be well-utilized during staff huddles and during onboarding, particularly when they were simple and included visual elements.

Other implementation strategies received greater criticism. Although the audit and feedback reports were noted to provide good insight into performance trends, champions reported issues with data transmission, interpretation and timeliness. Audit and provision of feedback is one of the most common and evidence-based implementation strategies (26); however, various factors can moderate their effectiveness, such as how the feedback is provided (27). Modifications were made to the reports to promote easier access, simplify metrics, and clarify data sources and categorization. At the mid-study in-person meeting, sites were also provided with a review of how to interpret data in the reports and encouraged to connect with the implementation science team to review the reports. Meeting with the implementation science team were also promoted more regularly to sites (e.g., at each learning collaborative meeting). Generation of data and overlap with internal processes were not as easily addressed, with no modifications identified to address these issues. However, the fact that many sites have their own audit and feedback systems may promote sustainability of this strategy past study termination and lead to enhanced sustainability of bundle implementation long-term. Use of the SCCM Connect message board was also low, particularly as the study proceeded, prompting a video resource containing instructions for easily accessing materials on SCCM Connect to be distributed to all sites. Posting frequency was also increased to encourage engagement. It remains to be seen if modifications to the strategies will effectively address the cited barriers, though continued data collection regarding fidelity to and use of implementation strategies will provide insight in the summative evaluation.

Many of the identified cross-cutting barriers were carried through from the formative evaluation (19) and spoke to larger issues outside the scope of the AIMS study. For instance, following the COVID-19 pandemic, staffing turnover in hospitals nationally is high (28, 29). Relatedly, staff report being overburdened and have a number of competing responsibilities to seeing sepsis patients and implementing sepsis bundles. The modifications made to the implementation strategies did not address these issues per se but added flexibility and options in how resources were allocated in addition to providing streamlined educational materials to reduce time burden and cognitive load. Cross-cutting facilitators of bundle use reported in interviews were primarily new and specific to the study processes. The most common was greater success in fostering leadership support for sepsis bundle implementation, which was cited primarily as a product of participating in the study. Champions were able to raise awareness and make a case for additional resources to support sustainable bundle implementation over time (e.g., hiring a dedicated sepsis coordinator). Although this is an artifact of the study process, the summative evaluation may provide a better understanding of the potential benefit of adding resources such as sepsis coordinators and provide generalizable knowledge to emergency departments as to whether this is a worthwhile investment. Respondents also reported a more supportive inter-organizational environment resulting from the collaborative learning and networking with other AIMS sites, which helped improve bundle implementation.

Overall, 14 adaptations were made to the study processes following the completion of this process evaluation and were tracked with the FRAME-IS. Given much of the feedback from interviewees was about the implementation strategies themselves, a large majority of the modifications were targeted to increase the acceptability, appropriateness, and feasibility of the implementation effort. In this hybrid type 2 effectiveness-implementation study, modifications were made under control of the research team at the study level and applied to all participating sites. The FRAME-IS is a relatively new method of documenting and categorizing modifications to the implementation process. Some research groups have indicated challenges in using the FRAME-IS (30), particularly for complex implementation strategies such as practice facilitation. However, its use was relatively straightforward in the current process evaluation. Others have noted that centralization of implementation efforts can help facilitate streamlined modification and documentation of implementation strategies (31), as was the case in the current evaluation. Notably, the process evaluation did not focus on changes made at the individual site level to support bundle implementation, which will likely offer more variation. Documentation and synthesis of individual site efforts is planned for the summative evaluation.

This process evaluation has a number of strengths, including the focus on an acute care setting, which is relatively understudied (16), and completion in the middle of the effort, which allows for real time modifications that can enhance the overall implementation. The study also spans 18 independent hospital sites, which helps support generalizability of the implementation strategies undertaken in the AIMS study, particularly given general satisfaction with the strategies.

Limitations of the process evaluation include no contextualization of implementation at the site level and thus, no accounting of individual site efforts to implement their assigned sepsis bundle. It also did not include objective measures of bundle implementation (e.g., completion of bundle elements in assigned timeline). Both of these factors are currently being tracked and will be reported in the summative evaluation to contextualize trial outcomes.

This process evaluation documents barriers and facilitators of implementation efforts to promote use of the 3-h and hour-1 sepsis bundles in emergency departments. Completion at the mid-point allowed for identification and implementation of modifications to address constructive feedback. Modifications made focused primarily on improving the acceptability, appropriateness, or feasibility of the implementation effort. Future directions of this research will be to understand if and how modifications made to the implementation effort of the sepsis bundle in the emergency department enhanced bundle implementation overall.

## Data Availability

The datasets presented in this article are not readily available because data is primarily qualitative in nature and therefore difficult to fully de-identify for data sharing. Requests to access the datasets should be directed to jacqueline_hayes@brown.edu.
